# Structural Relationships of Socioeconomic Factors Influencing Diet, Lifestyle Habits, Having a Dentist, and Health Factors That Impact Healthy Life Longevity for the Elderly

**DOI:** 10.3390/nu18030382

**Published:** 2026-01-23

**Authors:** Tanji Hoshi

**Affiliations:** Department of Urban Science, Tokyo Metropolitan University, 1-1 Minami-Osawa, Hachioji-City 192-0364, Japan; star@onyx.dti.ne.jp

**Keywords:** healthy life longevity, lifestyle including diet, socioeconomic status, structural relationship, having a dentist, confounding factors

## Abstract

**Background:** “Healthy Life Longevity” (a latent variable) is defined as the number of survival days, along with recommended subjective health and long-term care needs. This study aimed to clarify the structural relationships among several related factors. **Methods:** In September 2001, a postal survey using a self-administered questionnaire was conducted among 16,462 elderly residents of Tokyo. In a cohort study, 8162 individuals with confirmed survival after six years were examined. We analyzed data to evaluate the need for long-term care three years after the initial survey. Additionally, the number of days survived was calculated from the third year after the initial survey. Covariance structure analysis was used to explore the structural relationships. **Results:** The direct effects of lifestyle habits, including a healthy diet, dental care rather than physician care, and socioeconomic factors, were minimal in improving “Healthy Life Longevity.” However, a structural relationship was established: desirable lifestyles, including diet and dental care, were selected based on socioeconomic status, thereby influencing mental, physical, and social health and reducing disease incidence. This relationship ultimately enhanced “Healthy Life Longevity.” Socioeconomic factors were identified as confounders in the association between preferred lifestyle choices, including diet, and Healthy Life Longevity. The determination coefficient of “Healthy Life Longevity” is 83%. **Conclusions:** Although healthy longevity can be achieved by improving mental, physical, and social health, and reducing disease burden, the relevant structure is shaped by socioeconomic status. Additionally, socioeconomic status is associated with healthy longevity by facilitating the choice of a preferred lifestyle, including diet, and the selection of a dentist. Future randomized intervention studies focused on socioeconomic status should explore ways to promote healthy longevity.

## 1. Background

In 2024, the average life expectancy for men in Japan was 81.09 years, while for women, it was 87.13 years. The reason for Japan’s longevity is its high literacy rate and a world-class food culture that ensures access to a wide variety of ingredients.

Notably, women have held the No. 1 position worldwide for more than 40 years, whereas men’s ranking has fallen to No. 6 [1]. In countries such as Japan, with rapidly declining birth rates and an aging population, it is expected that “Healthy Life Longevity” (a latent variable) will not only extend lifespan but also help prevent the need for long-term care.

A new indicator introduced by the WHO in 2000, “Healthy Longevity,” measures the years people can live without health-related limitations, making it a significant health metric. However, in 2022, the gap between average life expectancy and healthy longevity in Japan was 8.49 years for men and 11.63 years for women [2]. Promoting “Healthy Life Longevity” helps stabilize healthcare costs and reduce the burden of nursing care. Total medical expenditure in fiscal 2024 was JPY 48.0 trillion, and total long-term care insurance expenditure in fiscal 2023 surpassed JPY 11.5 trillion [3]. Together, medical expenses and long-term care insurance premiums accounted for 51.6% of the national budget of JPY 115.5 trillion in fiscal 2025. This poses a significant financial challenge, as workers’ salaries in Japan have increased only modestly over the past 30 years.

Therefore, promoting “Healthy Life Longevity” is crucial because it not only enhances individuals’ quality of life and alleviates the burden on families but also reduces the country’s overall economic pressure.

Japan, facing an aging population and a declining birth rate, launched the “Healthy Japan Plan for Healthy Longevity 21” in 2000 [4]. The plan aimed to extend healthy lifespan by promoting a balanced diet and healthy behaviors, including oral hygiene and smoking cessation. However, it did not address the importance of socioeconomic factors.

Factors supporting survival are well-documented [5,6,7,8,9,10,11,12]. Maintaining healthy lifestyle habits, such as a balanced diet, is crucial for ensuring future survival [5]. Additionally, BADL (basic activities of daily living) and LIADL (instrumental activities of daily living) are recognized as physical indicators and measures of cognitive function that influence survival outcomes [6,7]. Other factors affecting survival include mental health, subjective health, and life satisfaction [8,9]. Social health, measured by the frequency of going outside and connection with neighbors, has also been associated with survival [10,11].

Socioeconomic factors are just as important because they lay the groundwork for maintaining healthy habits, such as a balanced diet, and for encouraging a positive lifestyle [12]. The income of older adults is linked to their educational attainment from approximately 50 years ago. In a follow-up study of older adults, higher annual incomes, supported by education, were associated with a longer life expectancy [12]. Additionally, it has been clarified that a structural framework exists in which mental health serves as the foundation, supporting physical health, and ultimately, social health is affected by socioeconomic factors, including annual income [12].

As a factor contributing to the increasing demand for long-term care, Komeji et al. [13] assessed the extent of long-term care needs among 532 older adults receiving home services. They found that a lower BMI, an indicator of undernutrition, was significantly associated with greater long-term care needs. At the same time, Ng et al. [14] and Lorenzo et al. [15] reported that lifestyle habits, such as exercise frequency and dietary choices, affect long-term care. Additionally, a strong link has been found between lifestyle habits, such as healthy eating, and socioeconomic factors in preventing cerebrovascular accidents, which often require long-term care. Previous studies [5,6,7,8,9,10,11,12,13,14,15] have shown that the maintenance of survival and the prevention of long-term care requirements have not been analyzed structurally. Clarifying the structural relationship can help focus on socioeconomic factors and related aspects to promote effective long-term care prevention, potentially improving preventive outcomes by assessing the level of long-term care needs. However, few studies on the structures related to preventing care dependency have been published outside of our research [16,17]. One reason is that although countries such as Germany, Sweden, Japan, and South Korea have implemented long-term care insurance systems, these systems are not universal. There are additional reasons for conducting multiple follow-up surveys over several years and for analyzing the data using covariance structure modeling.

Women typically require more long-term care than men and tend to live longer. As a result, long-term care needs pose a significant health challenge for women. The Ministry of Health, Labor and Welfare of Japan states that the higher rates of long-term care needs among women are mainly due to women exercising much less than men. Additionally, conditions such as cerebrovascular accidents, dementia, decreased bone density, and frailty are associated with increased long-term care needs. Although a healthy diet can support survival and reduce long-term care needs, limited information is available on background factors such as education and income, which primarily influence dietary requirements as a structural relationship, beyond our previous research [16,17].

This study aims to evaluate the survival span, subjective health, and long-term care needs of elderly residents in Tokyo’s suburbs as latent dependent variables. The dependent latent variable “Healthy Life Longevity” used in this paper is defined according to the WHO definition.

**Figure 1:**
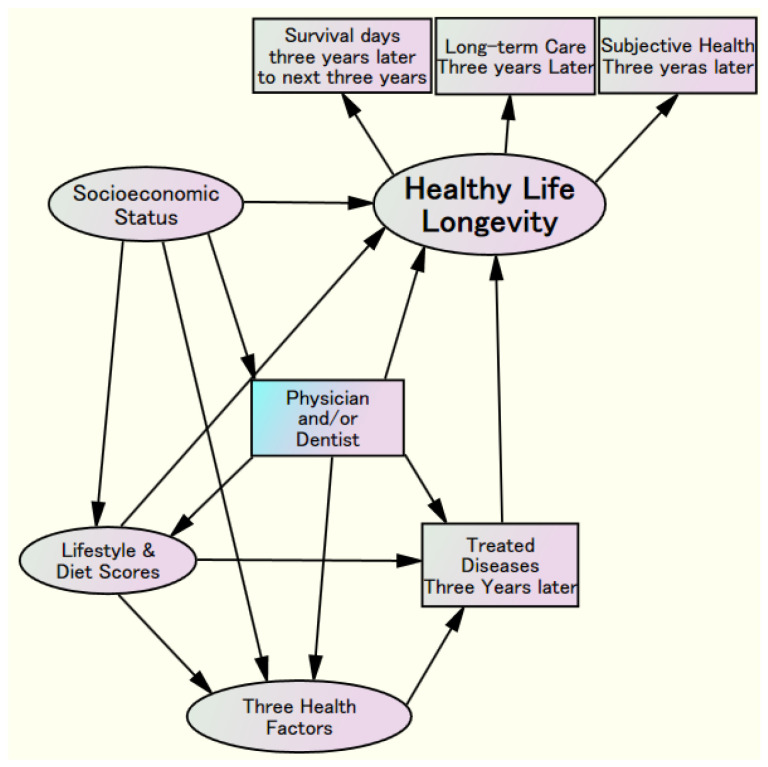
Hypothesis model of Healthy Life Longevity, socioeconomic status, lifestyle, and diet scores, three health factors, treated diseases, and having a physician and/or dentist.

It also examines how lifestyle habits, including diet, relate to physical, mental, and social health. Furthermore, this study seeks to clarify the structural relationships among socioeconomic factors, health components, and related diseases. Additionally, the findings are expected to provide stronger scientific evidence for effective, systematic strategies to prevent the development of long-term care needs, especially among women. The structural hypothesis model, showing the relationships among “Healthy Life Longevity,” including survival duration and self-assessed health, and the level of long-term care required, along with related factors, is presented in Figure 1.

What makes this study superior to our previous research [12,16,17] is the introduction of subjective health perception into the study of “Healthy Life Longevity.” In addition, dietary habits associated with survival were incorporated into analyses of survival-related eating frequency and the number of diseases under medical treatment after three years. In addition, height was introduced as an observational variable among socioeconomic factors. The reason is that height is evidence of strong family support for childhood nutrition [18,19]. Ultimately, we sought to increase the coefficient of determination for “Healthy Life Longevity,” a newly defined latent dependent variable.

## 2. Research Methods

### 2.1. Research Subjects

In September 2001, we surveyed all elderly residents aged 65 or older living at home in suburban Tokyo, particularly in Tama City. Of the 16,462 seniors in these areas, 13,066 (a response rate of 79.4%) provided informed consent and returned the self-administered questionnaire by mail. In September 2004, we sent the same questionnaire to these respondents again. Among them, 505 had moved away, 914 had died, and 4089 did not respond. All participants were followed until 31 August 2007. A total of 8162 individuals, including 3851 men and 4311 women aged 65 to 84 at the time of the initial survey, were included in the analysis to assess the need for long-term care three years later. The number of survival days was calculated from year three through the next three years, based on data stability.

### 2.2. Research Fields

The cities included in this study developed as commuter towns in the Tokyo metropolitan area to accommodate the influx of workers and their families during Japan’s rapid economic expansion in the 1970s and 1980s. Most residents are middle-class, and by 2000, the population reached 145,862, with 11.1% aged 65 or older. A systematic survey was conducted under an agreement between the mayor of the local government and the president of Tokyo Metropolitan University to develop effective health policies that support Healthy Life Longevity.

### 2.3. Survey Details

(1) Socioeconomic factors

These surveys used validated, standardized questions to assess health status and lifestyle. In 2001, socioeconomic status was determined by education level and annual income. Educational backgrounds were categorized into three groups: high school graduates, junior college graduates, and university graduates, excluding non-respondents. The annual income of married couples in 2001 was categorized into four groups: less than JPY 1 million (less than USD 6667, given the exchange rate of 1 USD = JPY 150), less than JPY 3 million, less than JPY 7 million, and more than JPY 7 million. In addition to education, marital status, and age, we included height to analyze socioeconomic status. A certain level of height growth indicates a healthy and prosperous childhood. Height has been recognized as a valid and highly accurate indicator of survival after half a century [18,19]. According to Jousilahti et al. [18], a 15-year follow-up study of 31,199 adult residents in eastern Finland found that shorter stature was associated with higher overall mortality. Similarly, we tracked the survival of 13,460 older adults in the suburbs of our country over three years. A similar study by KO et al. in South Korea also reported a significant reduction in survival among individuals with short stature [19].

(2) Lifestyle and dietary characteristics

Healthy lifestyle factors are habits strongly associated with the number of days survived over six years. Habits that significantly enhanced survival included drinking daily, quitting smoking (even in the past), sleeping fewer than 9 h per night, exercising at least once a week, and maintaining a BMI above 20 (i.e., not losing weight). As a result, each of these five healthy habits is scored from 0 to 5, with higher scores indicating more desirable habits, as shown in the previous study [5] and based on our study [16].

With respect to dietary habits, the frequency of food intake is strongly associated with survival over the next three years. Eating eggs more than once or twice per week is associated with significantly improved survival compared with those who do not eat eggs. Similarly, consumption of blueback fish, vegetables, fruits, salted foods, and oil-based dishes follows the same pattern. Eating dairy products more than three days per week is associated with improved survival compared with those who do not consume dairy. Following the approach of the previous study by Berkman et al. [20], a food score was assigned, ranging from 0 to 7 points.

(3) Mental, physical, and social health

Among the health factors examined, the physical, mental, and social components were selected in accordance with the WHO definition of health. Physical health parameters included basic activities of daily living (BADL) [6] and instrumental activities of daily living (IADL) [7]. Three questions assessed the BADL score: “Can you go to the bathroom alone?” “Can you take a bath by yourself?” “Can you go out by yourself?” Each person received one point for each function they could perform; the total score ranged from 0 to 3, with higher scores indicating greater ability to perform basic activities. The IADL score assesses five instrumental activities of daily living, including: “Can you go shopping by yourself?” “Can you cook your own meals every day?” “Can you deposit and withdraw money from your bank account?” “Can you fill out insurance and pension documents?” “Can you read books and newspapers?” The IADL score is similar to the BADL score, with a total range of 0 to 5; higher scores indicate greater ability to perform instrumental activities of daily living.

Mental health is assessed through self-report using subjective health indicators. The question is, “Do you consider yourself healthy?” with four options: very healthy, moderately healthy, not so healthy, and not healthy at all [8]. The life satisfaction question asks, “Are you satisfied with your daily life?” and provides three options: very satisfied, moderately satisfied, and dissatisfied [9]. Outdoor activities and interactions with neighbors were evaluated as indicators of social health. For the question about how often one goes outside, “How often do you go outside, including in the neighborhood?” responses ranged from less than once a month to more than three or four times a week [10]. Communication with neighbors was assessed by asking, “How often do you communicate with friends and neighbors?” Responses were categorized as rarely (about once a month), 3–4 times a week, or almost every day [11].

(4) Diseases being treated

The five diseases most strongly associated with decreased survival after six years were hypertension, cerebrovascular disease, diabetes, heart disease, and liver disease. Without accounting for disease severity, the number of treated diseases three years later was scored on a 0–5 scale.

(5) Long-term care needs

Japan’s Ministry of Health, Labor, and Welfare created a publicly accessible tool to assess nursing care levels. The assessment includes six tiers, ranging from the least severe (requiring minimal support) to the most severe (requiring extensive care and assistance). In the analysis, respondents who did not receive care were assigned a score of 0, with the lowest severity level at 1 and the highest at 6. In this article, being bedridden is defined but considered equivalent to requiring long-term care. The survey was conducted three years later.

### 2.4. Analysis Methods

The relationship between dependent and explanatory variables was assessed using the chi-squared test or Kendall’s τ test. For continuous variables, comparisons were made with one-way analysis of variance. To clarify the structure of a hypothetical model with latent variables, we applied covariance structure analysis [21,22]. The latent variables were identified through exploratory factor analysis with varimax rotation and maximum likelihood estimation. In structure analysis, the model’s goodness-of-fit indices included the NFI (Normed Fit Index), the IFI (Incremental Fit Index), and the RMSEA (Root Mean Square Error of Approximation) [21,22]. All estimates were standardized, and statistically significant differences were considered as those with *p*-values < 0.05. Data analysis was performed using the Statistical Package for the Social Sciences (SPSS) and AMOS version 28.0 (IBM, New York, NY, USA).

## 3. Results

### 3.1. Survey Subjects

A total of 8162 people participated in the survey, including 3851 men and 4311 women. The participants’ attributes and characteristics were described in previous studies as a basic distribution and correlations among them [12,16,17,23].

### 3.2. The Relationship Between the Level of Need for Long-Term Care After 3 Years and Survival Days, Along with Each Explanatory Factor

The relationship between long-term care needs three years after and survival status three years later—covering the subsequent three years—was statistically significant for all 13 explanatory factors. In other words, higher annual income, greater height, and 11 other favorable factors helped maintain care needs over three years, and survival during this period was significantly better for both sexes [12,16,17,23].

Although all 13 explanatory factors were associated with care needs and survival, the correlations among these factors, as well as their relationships and structures, remain unclear. Understanding the cause-and-effect relationships involved, especially confounding factors, is crucial. Therefore, we used covariance structure analysis to explore this.

Among the seven food groups, the group that ate salty foods more than once a week had more survival days than the group that did not. Specifically, both men and women who consumed salted products more than once a week lived significantly longer than those who did not. Consequently, the results of the one-way analysis of variance for unary configuration, the frequency of salted product intake, and survival days for both sexes are shown in Table 1. The higher the preferred food score, the greater the reduction in long-term care after 3 years, and it also significantly increased their survival days over the following 3 years [23]. However, this study did not measure the intake, energy, or nutrient content of each food.

Details on the statistically significant relationships between maintenance of survival and survival days, and between the degree of care required after 3 years and survival days, have been reported in previous studies [12,16,17,23]. Therefore, the related table is omitted from this paper.

### 3.3. Structural Correlation Between the Latent Variable “Healthy Life Longevity” and Explanatory Factors

(1) Applying exploratory factor analysis to discover hidden variables in covariance structure analysis

To clarify the latent variables, we conducted a factor analysis using the maximum likelihood method and Promax oblique rotation. The study results showed that the first factors were [height] ([ ] indicating observed variables), [educational background], and [yearly income]; the scores for each factor were 0.649, 0.628, and 0.439, respectively, as a “Socioeconomic Status.” The second factor comprised [BADL], [IADL], and [going outside]; the factor scores were 0.729, 0.450, and 0.369, respectively. The third factor comprised [subjective health], [life satisfaction], and [contact with the neighbors]; the scores for each factor were 0.559, 0.499, and 0.420, respectively. The [Treated Diseases] and [Physician and/or Dentist] were treated as observed variables; the factor scores were −0.445 and −0.334, respectively. The fifth factor comprised [lifestyle score] and [diet scores]; the scores for each factor were 0.381 and 0.324, respectively, categorized as “Lifestyle and Diet Scores.” Additionally, the sum of the squares of these six factors totaled 36.3% (Table 2).

(2) Structure Model with “Healthy Life Longevity” as a Dependent Variable

Because of the same survey period, the “Socioeconomic Status,” “Lifestyle and Diet Scores,” and “Three Health Factors” items can function as both causes and effects. We analyzed the direction and strength of structural relationships across all combinations of variables. Using the correction metrics, we identified the best-fitting model. Based on the literature [21,22], we selected the model with the highest standardized estimate.

**Figure 2:**
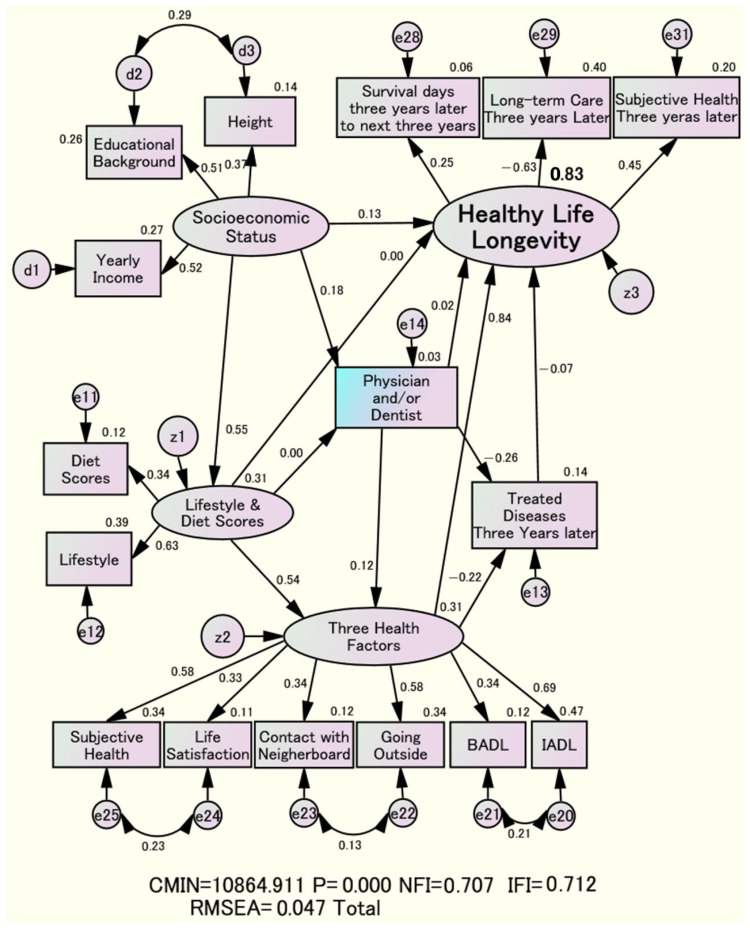
Structural relationship among “Three Health Factors” supporting “Healthy Life Longevity,” “Socioeconomic Status,” “Lifestyle and Diet Scores,” and [Physician and/or Dentist], and [Treated Diseases].

As a result, “Socioeconomic Status,” “Three Health Factors,” and “Lifestyle and Diet scores” were identified as potential explanatory variables for “Healthy Life Longevity.” Ultimately, the model fit indices in Figure 2 indicated a good fit, with NFI = 0.707, IFI = 0.712, and RMSEA = 0.047 [21,22]. Additionally, [Treated Diseases], measured three years later, was included as an explanatory variable. Furthermore, all relationships between latent and observed variables were statistically significant in the Wald test. The overall effect comprised both direct and indirect relationships with the dependent latent variable “Healthy Life Longevity” (Table 3).

**Figure 3:**
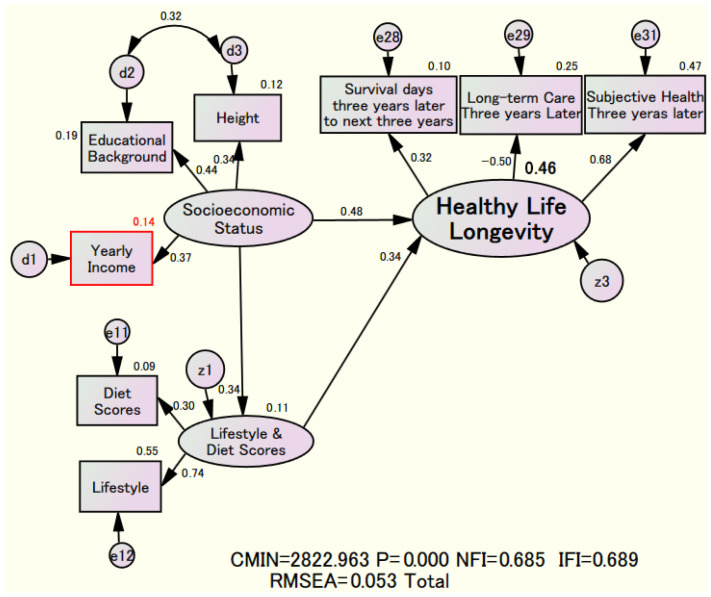
Structural relationship among “Socioeconomic Status,” “Lifestyle and Diet Scores,” and “Healthy Life Longevity”.

**Figure 4:**
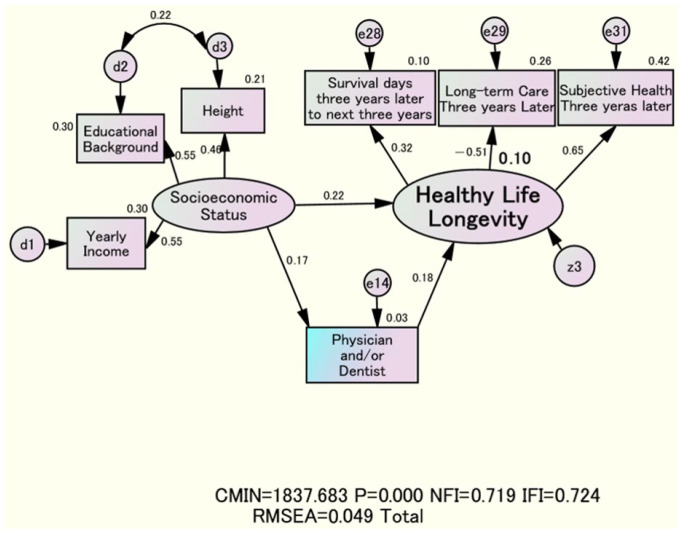
Structural relationship among “Socioeconomic Status,” [Physician and/or Dentist], and “Healthy Life Longevity”.

The ellipsometer shown in Figure 2, Figure 3 and Figure 4 depicts the latent variable; the rectangle represents the observed variable, and the circle indicates the error variable. The numbers above the arrows are normalized estimates with absolute values of 1 or less, indicating the strength of the relationships. The number inside the oval variable is the coefficient of determination.

(3) Structure effect of each explanatory variable on “Healthy Life Longevity.”

The most significant direct effects on “Healthy Life Longevity” occurred “Three Health Factors,” three years ago, with standardized estimates of 0.84 directly. “Three Health Factors” directly and most significantly impacted the maintenance of “Healthy Life Longevity” (Figure 2).

Conversely, the direct effects of “Socioeconomic Status” on “Healthy Life Longevity” were at 0.13. The direct effects of “Lifestyle and Diet Scores” on “Healthy Life Longevity” were almost zero and statistically nonsignificant.

Additionally, the “Three Health Factors” had the most significant overall effect, with a combined direct and indirect effect on “Healthy Life Longevity” of 0.85. This was followed by “Lifestyle and Diet Scores” at 0.46 and “Socioeconomic Status” at 0.41. In essence, higher “Socioeconomic Status” can support “Lifestyle and Diet Scores,” and choosing a dentist rather than a physician can improve the “Three Health Factors” and prevent [Treated Diseases], thereby increasing overall “Healthy Life Longevity.” The direct effect of the “Three Health Factors” on [Treated Diseases] was −0.22 (Figure 2, Table 3).

The coefficient of determination for “Healthy Life Longevity,” considering all explanatory factors, was 83% (Figure 2). The related structures showed a nearly consistent pattern across genders.

#### 3.3.1. Relationship Structure from “Socioeconomic Status” to “Lifestyle and Diet Scores” and “Three Health Factors”

The direct effect of “Socioeconomic Status” on “Lifestyle and Diet Scores” is 0.55. The coefficient of determination for the “Lifestyle and Diet Scores” is 31%. The effect from “Lifestyle and Diet Scores” to “Three Health Factors” is 0.54. The coefficient of determination for the “Three Health Factors” is 31%. Additionally, the indirect effect of “Socioeconomic Status” on the “Three Health Factors” via “Lifestyle and Diet Scores” is 0.30 (i.e., 0.55 × 0.54).

Figure 3 illustrates the relationship between “Socioeconomic Status” and “Lifestyle and Diet Scores” that support “Healthy Life Longevity.” The results differ from those in Figure 2. Specifically, the direct effect of “Lifestyle and Diet Scores” on “Healthy Life Longevity” is 0.34.

In contrast, the estimates in the final model in Figure 2 are almost zero. This indicates that the main influence of “Lifestyle and Diet Scores” on “Healthy Life Longevity” is primarily driven by the recommended “Socioeconomic Status.” Additionally, “Socioeconomic Status” may be a confounding variable because “Lifestyle and Diet Scores” are not mediators of the effect of “Socioeconomic Status” on “Healthy Life Longevity.” This indicates that “Lifestyle and Diet Scores” have no impact on “Socioeconomic Status,” making it difficult to control for [24]. The key finding of this study is that “Socioeconomic Status” is a confounding factor between “Lifestyle and Diet Scores” and “Healthy Life Longevity,” as shown in Figure 2 and Figure 4.

#### 3.3.2. Relationship Structure from [Treated Diseases] to “Healthy Life Longevity”

Three years ago, the strongest direct contributor to the prevention of [Treated Diseases] was [Physician and/or Dentist], with an estimated value of −0.26. Choosing a dentist rather than a doctor reduces the number of diseases that must be treated, ultimately leading to “Healthy Life Longevity” (Figure 2).

#### 3.3.3. Relationship Structure from [Physician and/or Dentist] to “Healthy Life Longevity”

The standardized estimate of the effect of [Physician and/or Dentist] on “Healthy Life Longevity” was only 0.02 in Figure 2, compared to 0.18 in Figure 4. In this way, the relationship between [Physician and/or Dentist] and “Healthy Life Longevity” also suggests that “Socioeconomic Status” may be a confounding factor.

The overall effect of [Physician and/or Dentist] on “Healthy Life Longevity” was 0.13, equivalent to the overall effect of “Lifestyle and Diet Scores” of 0.12. In addition, compared with the total effect of 0.41 for “Socioeconomic Status,” the shared effect was approximately 29.3% (0.12/0.41).

## 4. Discussion

### 4.1. Structure of Lifestyle, Including Diet Related to Healthy Life Longevity

The direct effect of “Lifestyle and Diet Scores” on “Healthy Life Longevity” was minimal. Instead, “Socioeconomic Status” laid the foundation, and the recommended “Lifestyle and Diet Scores” positively influenced the subsequent “Three Health Factors,” which contributed to a decrease in the number of [Treated Diseases] on the path to “Healthy Life Longevity.” The coefficient of determination for “Healthy Life Longevity,” considering all explanatory factors, was 83% (Figure 2).

Although a healthy lifestyle, including an advisable diet, can help improve survival days through simple relational analysis, we should focus on the fundamental fact that the structure of survival depends on increasing the “Lifestyle and Diet Scores” and “Three Health Factors,” and on decreasing the [Treated Diseases], with “Socioeconomic Status” as a confounding factor.

In this study, compared to the previous original paper [16] in 2018, which suggested that socioeconomic factors could confound the relationship between lifestyle habits and Healthy Life Longevity, nearly exact reproducibility was achieved by adding height to socioeconomic factors, reworking the diet score, and including the observation variable of the number of diseases treated after 3 years. Additionally, the same confounding factors were identified when the number of days of survival was used as the dependent observed variable [23].

Confounding factors are variables associated with both the cause and the outcome that are not intermediate steps in the causal pathway [24]. Shibata et al. [25] surveyed individuals aged 70 or older (J-AHEAD) in Japan and, after controlling for confounding factors, reported that paid work and other daily activities were associated with a lower risk of death. Apart from the authors’ previous research reports [12,16,17], there appear to be no other studies analyzing the related structure, including socioeconomic status and “Healthy Life Longevity.”

The diets in this study were examined only for the frequency of seven foods associated with survival maintenance. In the future, a detailed analysis of food calories and ingredients is expected.

In the United States, a high-quality randomized multiple risk factor intervention trial (MRFIT) was conducted to identify a high-risk group among 350,000 participants and then randomly assign them to receive either a behavioral intervention or standard care to maintain survival through behavior change. In particular, the MRFIT study helped participants quit smoking, and blood pressure and total cholesterol levels were significantly reduced after 7 years of follow-up. However, there was no difference in survival between the intervention and control groups [26]. Additionally, a similar intervention follow-up study on behavior change was conducted in Finland. In 1974, 1222 people who were clinically healthy but at risk for medical examination were selected from 3490, and an intervention group of 612 and a control group of 610 were randomly assigned. As a result, over 15 years, 67 people died in the intervention group and 46 in the control group. The group that received education and was encouraged to change their behavior experienced a statistically significant increase in overall mortality [27].

Well-known prior research by L. Breslow et al. [5] showed that favorable lifestyle habits are strongly associated with longevity. We should consider the importance of socioeconomic factors that support their maintenance without requiring behavior change in adulthood, as indicated by the study’s results. It was suggested that the significance of health support based on height [18,19], which mainly depends on the family’s desired diet, was important in the educational background of about half a century ago and in early childhood and school years. Therefore, it is important to recognize the importance of health support, especially an advisable diet during childhood.

Based on previous studies [18,19,26,27], home education is an important foundation for the development of lifestyle habits, including dietary preferences. It was suggested that it is important to eliminate the need for behavior change toward a preferred lifestyle after adulthood.

### 4.2. Structure of Having a Dentist Rather than a Physician for Maintaining a “Healthy Life Longevity”

Supported by socioeconomic factors, the tendency to see a dentist rather than a physician was associated with the three health factors being desirable and with disease control during treatment, ultimately indicating that a related structure was associated with healthy longevity. However, the presence of only a family dentist does not directly ensure healthy longevity, and, as shown in the recommended lifestyle, socioeconomic factors and lifestyle habits were also suggested as confounders (Figure 2 and Figure 4). We reported that the 6-year cumulative survival rate among individuals with a family dentist is significantly higher than that among individuals without one [28]. However, the analysis did not account for the relevant structure, including background socioeconomic factors.

The interpretation that maintaining family dentistry will lead to a longer life expectancy is incorrect. It was suggested that attention be paid to related structures that are favorable for socioeconomic conditions, which only family dentists can provide, resulting in fewer diseases being treated and no need for physician consultation. We should prioritize socioeconomic factors to ensure access to dentists, rather than physicians, to live much longer. Reproducibility is required.

According to Zhao et al. [29], oral hygiene can prevent pneumonia. However, the significance of the socioeconomic factors that should be in the background remains unclear. In a randomized interventional study with a control group on oral hygiene among institutional residents, Yoneyama et al. reported that pneumonia incidence decreased and survival improved [30]. Intervention studies that favor oral hygiene suggest that it is associated with greater food abundance, particularly among individuals with favorable socioeconomic backgrounds, and with recommended self-oral hygiene practices.

### 4.3. The Importance of the Three Health Factors for Maintaining a Healthy Life Longevity

According to this study, the three strongest determinants of “Healthy Life Longevity” were mental, physical, and social health. However, these three health factors were found to reflect a related structure defined by lifestyle habits, including diet and greater freedom in choosing dentists. The basis for these relational factors was socioeconomic factors. In this way, this study shows that socioeconomic factors are not directly linked to “Healthy Life Longevity”; rather, they underpin lifestyle habits and the Three Health Factors and decrease the prevalence of treated diseases.

Low BMI, an indicator of undernutrition, was reported by Komeji et al. to be associated with a reduced need for long-term care [13]. In this study, we confirmed Komeji et al.’s findings through a follow-up study that included socioeconomic status.

It has been reported that dementia increases the need for subsequent care [31]. However, this study did not include reliable measures such as the Mini-Mental State Examination (MMSE), which is used to diagnose cognitive function medically. Nonetheless, this study showed that IADL, which is similar to cognitive function, is a crucial factor in determining the need for nursing care after three years. Therefore, in addition to future surveys, it is necessary to include measures of cognitive function, such as the MMSE [31].

It has become clear that the number of diseases to be treated, including cerebrovascular accidents, influences Healthy Life Longevity. Nevertheless, the severity of these diseases and the specifics of treatment remain unclear. This remains a topic for future research.

### 4.4. Socioeconomic Status Underlying Healthy Life Longevity

Height is recognized as an observable indicator of socioeconomic status that can influence Healthy Life Longevity. It is estimated that height is associated with a higher survival rate for more than half a century, not only because of better parental genetics but also because of attentive family support after birth [18,19]. Postnatal parental influences on children are often referred to as “memes” and are distinct from genetic factors [32]. By applying the concept of “selfish genes” (the “meme”), introduced by Richard Dawkins in 1976, parental influence can be viewed as a controllable meme rather than an uncontrollable gene. While genetic factors are not controllable, memes can be changed. Therefore, family support for postnatal health should be a core component of family education, particularly regarding an appropriate diet.

Socioeconomic status underpins a healthy family, spans multiple generations, and supports health maintenance. Specifically, it is expected that intergenerational skills will be passed down and developed to ensure a reliable food supply and promote healthy lifestyle choices. This underscores the need for health support focused on building family skills.

Socioeconomic status was identified as the primary factor in Healthy Life Longevity. However, controlling for factors such as age, height, and educational background is difficult. Among these, annual income is the easiest to manage [12].

It has also been shown that higher annual incomes, supported by higher education, are linked to longer survival. However, there is a threshold in this relationship. Specifically, the threshold was JPY 4.5 million for the annual household income of elderly couples living in urban areas. For elderly individuals living in rural areas, the income threshold was JPY 2.5 million [12]. At the same time, items such as economic living orientation and savings amount could also be added under “Socioeconomic Status.” Future research aims to improve the external validity of this study.

### 4.5. Future Research Issues

In this study, we examined the structure of “Healthy Life Longevity,” focusing on the necessary level of care after three years and subsequent survival maintenance, and advisable subjective health as the observed variables. Future research should examine “Three Health Factors,” “Socioeconomic Status,” and “Lifestyle Diet Scores” across different periods to better elucidate the causal structure rather than merely the structural association.

There are also issues with survey indicators. All explanatory factors, except long-term care and days alive, used to measure the dependent latent variables, rely on self-reports. Future research should include evaluation indicators that use objective data from medical examinations, dietary intake, and counts of calories and fine minerals to assess structural relationships.

Additionally, to improve the coefficient of determination for “Healthy Life Longevity,” it is important to increase the coefficients for both “Lifestyle and Diet Scores” and “Three Health Factors.” Among lifestyle habits, not only exercise frequency but also intensity and duration should be specified; in food surveys, not only food items and their frequency but also calories and nutrients should be included; and MMSE scores should be used to assess cognitive function. A study of 1485 patients admitted to acute geriatric wards in Germany found that daytime sleepiness was associated with in-hospital falls among elderly patients [33]. In this study, we focused on sleeping less than 9 h as a preferred lifestyle; in the future, we plan to add drowsiness as a new survey item to evaluate “Healthy Life Longevity.”

Additionally, this study reported that aging and health awareness are associated with maintaining healthy lifestyle habits and the Three Health Factors, and that these factors are linked to survival, as shown below [34,35,36]. Therefore, this should improve the coefficient of determination for “Healthy Life Longevity.” A positive outlook on the future is associated with a lower risk of long-term care [34]. Tully-Wilson et al. also emphasized the importance of viewing aging positively and demonstrated the predictive validity of survival and life satisfaction [35]. Positive attitudes toward retirement have also been linked to higher survival rates [36].

Sapporo et al. [37] conducted an intervention study focusing on the subjective health of older adults in the community. The findings showed that health education interventions aimed at improving subjective health over 18 months were more effective than the control group in preventing institutionalization and death. Therefore, future practical efforts to reduce the need for care among older adults should emphasize the importance of maintaining subjective health. It is expected that health support will be provided with the understanding that subjective health is influenced by positive lifestyle habits and, as a foundation, by socioeconomic status.

According to the WHO-recommended community program, various supportive elements should be included, such as a living environment that encourages physical activity, promotes healthy eating habits, fosters psychological well-being, and supports social engagement while taking socioeconomic status into account [38]. In the future, the analysis is expected to account for the characteristics of the local community environment and government health initiatives that promote social interaction.

The “social prescription” initiative in the United Kingdom [39] aims to connect many people with non-medical social resources to address social problems and improve their health. It was expected that this initiative would improve the means of addressing the social determinants of health. In Japan, in 2020, ‘social prescription’ was taken up as a policy issue in the ‘Framework Policy’ (the basic policy for economic and fiscal management and reform). According to Kumakawa et al. [40], the use of social prescriptions is expected to strengthen the coordination function of local resources.

The U.S. Department of Health and Human Services then reported on Healthy People 2030 [41]. In this report, five factors are presented as health determinants. The first is Economic Stability, followed by Education Access, Health Care Access and Quality, Neighborhood and Built Environment, and Social and Community Context. In addition, we should examine the support environment for these factors.

This survey found that the need for nursing care was higher among older women. However, there was no statistically significant difference between genders as the main factor influencing the amount of care required. Future research should explore genetic factors that allow women to live longer than men, the impact of female hormones on bone density loss, and personal details like falls and fractures caused by muscle weakness.

As medical care advances, long-term hospitalization, which often emphasizes rest, may increase demand for long-term care. Kurimori et al. reported that a higher number of hospital beds per capita in a prefecture correlates with a significantly higher proportion of long-term care certifications [42]. According to the 2023 patient survey by the Ministry of Health, Labor, and Welfare of Japan, the average hospital stay was 29.3 days [43]. In this way, patients stay hospitalized about 3 times longer than the OECD average of roughly 8 days worldwide. This suggests that analyses should account for the high number of hospital beds per capita and the tendency toward longer hospital stays.

Therefore, future surveys should include analyses of individuals’ hospitalization histories and treatment status to better estimate “Healthy Life Longevity,” thereby increasing the coefficient of determination. Additionally, future surveys should ask about the presence or absence of fractures and hospitalizations. Specifically, clarifying this research’s focus can be achieved by categorizing individuals who have undergone femoral and knee replacement surgeries, as well as those with fractures that are easier to treat. Since serious injuries like fractures can often lead to long-term care needs, but such dependence can improve in less than six months, this classification will help better understand long-term care requirements.

Additionally, as a future research topic, it is important to improve the external validity of the study results by conducting intervention studies that include a control group, account for factors such as socioeconomic status, and ensure the reproducibility of the randomization process for sample selection.

## 5. Conclusions

Although healthy longevity can be achieved by improving mental, physical, and social health and reducing disease burden, the relevant structure is shaped by socioeconomic status. Additionally, socioeconomic status is associated with healthy longevity by facilitating the choice of a preferred lifestyle, including diet, and the selection of a dentist. Future randomized intervention studies focused on socioeconomic status should explore ways to promote healthy longevity.

## Figures and Tables

**Table 1:** One-way ANOVA between frequency of intake of salted products and days of survival by sexes.

Sex	(I) Salty Food	(J) Salty Food	Survival Days (I–J)	*p* Value	95% Confidential	
					Upper	Lower
Men	Almost every day	5~6 day/week	7.048	1.000	−14.347	28.443
		3~4 days/week	3.166	1.000	−14.327	20.659
		1~2 days/week	−1.856	1.000	−18.567	14.855
		not take	33.391	0.023	2.593	64.188
	5~6 days/week	3~4 days/week	−3.881	1.000	−26.734	18.971
		1~2 days/week	−8.904	1.000	−31.163	13.355
		not take	26.343	0.302	−7.784	60.470
	3~4 days/week	1~2 days/week	−5.022	1.000	−23.563	13.518
		not take	30.225	0.077	−1.602	62.052
	1~2 days/week	not take	35.247	0.016	3.843	66.651
Women	Almost every day	5~6 day/week	−9.847	0.953	−26.422	6.728
		3~4 days/week	2.453	1.000	−11.642	16.548
		1~2 days/week	4.395	1.000	−9.914	18.704
		not take	38.653	0.000	12.798	64.509
	5~6 days/week	3~4 days/week	12.300	0.634	−6.304	30.904
		1~2 days/week	14.242	0.331	−4.524	33.009
		not take	48.500	0.000	19.936	77.065
	3~4 days/week	1~2 days/week	1.942	1.000	−14.675	18.560
		not take	36.201	0.002	9.000	63.402
	1~2 days/week	not take	34.258	0.004	6.946	61.571

**Table 2:** Results of exploratory factor analysis.

	Socioeconomic Status	Three Health Factors	Three Health Factors	Physician and/or Dentist	Life Style and Diets
**Height**	0.649	0.069	-0.025	0.010	−0.101
**Educational Background**	0.628	0.049	-0.036	0.079	0.128
**Yearly Income**	0.439	−0.005	0.153	0.015	0.209
**IADL**	0.034	0.729	0.186	0.190	0.159
**BADL**	0.029	0.450	0.019	0.024	0.034
**Going Outside**	0.070	0.369	0.281	−0.034	0.204
**Subjective Health**	0.118	0.184	0.559	0.477	−0.018
**Life Satisfaction**	0.045	0.073	0.499	0.076	0.069
**Contact with the Neighbors**	−0.080	0.095	0.420	−0.029	0.246
**Treated Diseases**	0.097	−0.071	−0.060	−0.445	−0.107
**Physician and/or Dentist**	−0.121	−0.009	−0.014	−0.334	−0.038
**Lifestyle Scores**	0.080	0.066	0.207	0.125	0.381
**Diet Scores**	0.054	0.097	0.036	0.056	0.324

**Table 3:** Direct and total effects on “Healthy Longevity” by “Three Health Factors” and “Socioeconomic Status” by sexes.

		Men	Women	Total
Standardized Direct Effect	“Socioeconomic Status” ⇒ “Healthy Life Longevity”	0.05	0.15	0.13
	“Socioeconomic Status” ⇒ “Lifestyle and Diet Scores”	0.53	0.66	0.56
	“Lifestyle and Diet Scores” ⇒ “Healthy Life Longevity”	0.00	0.00	0.00
	“Lifestyle and Diet Scores” ⇒ “Three Health Factors”	0.60	0.53	0.54
	“Lifestyle and Diet Scores” ⇒ [Physician and/or Dentist]	0.07	−0.03	0.00
	“Three Health Factors” ⇒ “Healthy Life Longevity”	0.88	0.82	0.84
	“Three Health Factors” ⇒ [Treated Diseases]	−0.23	−0.22	−0.22
	“Lifestyle and Diet Scores” ⇒ [Physician and/or Dentist]	0.07	−0.03	0.00
	[Physician and/or Dentist] ⇒ [Treated Diseases]	−0.24	−0.28	−0.26
	[Physician and/or Dentist] ⇒ “Healthy Life Longevity”	0.04	0.04	0.02
	[Physician and/or Dentist] ⇒ “Three Health Factors”	0.08	0.13	0.12
Standardized Total Effect	“Socioeconomic Status” ⇒⇒ “Three Health Factors”	0.34	0.37	0.32
	“Socioeconomic Status” ⇒⇒ [Treated Diseases]	−0.12	−0.13	−0.12
	“Socioeconomic Status” ⇒⇒ [Physician and/or Dentist]	0.17	0.18	0.18
	“Socioeconomic Status” ⇒⇒ “Healthy Life Longevity”	0.35	0.47	0.41
	“Lifestyle and Diet scores” ⇒⇒ [Treated Diseases]	−0.16	−0.11	−0.12
	[Physician and/or Dentist] ⇒⇒ “Healthy Life Longevity”	0.11	0.14	0.13
	“Lifestyle and Diet scores” ⇒⇒ “Healthy Life Longevity”	0.54	0.44	0.46
	“Three Health Factors” ⇒⇒ “Healthy Life Longevity”	0.89	0.84	0.85

“ ”: Latent variable; [ ]: Observed variable; ⇒: Direct Effect; ⇒⇒:Total Effect.

## Data Availability

The analytical data from this study are available from the UMIN SYSTEM in Japan as an open system (https://upload.umin.ac.jp/cgi-bin/fileshare/upload.cgi?DELETE=1&on=589518, accessed on 1 July 2025). Personal registration is required for data specifications. Additionally, analytical data and materials can be obtained from the first author via the following email address: star@onyx.dti.ne.jp (T. Hoshi).
